# Delayed diagnosis of juvenile hemochromatosis due to missed ferritin testing in a case of hypogonadotropic hypogonadism

**DOI:** 10.1210/jcemcr/luag159

**Published:** 2026-06-09

**Authors:** Purnima Agarwal, Ambrish Mithal, Komarakshi Balakrishnan, Murali Krishna Tanguturu, Suresh Rao Kemundel Genny

**Affiliations:** Department of Endocrinology, Vardhman Mahavir Medical College & Safdarjung Hospital, New Delhi 110029, India; Institute of Endocrinology and Diabetes, Max Super-Specialty Hospital, New Delhi 110017, India; Institute of Heart and Lung Transplant and Mechanical Circulatory Support, MGM Healthcare, Chennai 600029, India; Institute of Heart and Lung Transplant and Mechanical Circulatory Support, MGM Healthcare, Chennai 600029, India; Institute of Heart and Lung Transplant and Mechanical Circulatory Support, MGM Healthcare, Chennai 600029, India

**Keywords:** juvenile hemochromatosis, hypogonadotropic hypogonadism, cardiomyopathy, insulin-dependent diabetes mellitus, genetic testing, polycystic ovary syndrome

## Abstract

Juvenile hemochromatosis (JH) is a rare disorder caused by iron metabolism errors that lead to severe iron loading and organ failure in young adults before 30 years of age. We report a challenging case of a 26-year-old female whose diagnosis was delayed by nearly a decade due to deceptively nonspecific symptoms. She initially presented with oligomenorrhea at age 18 and was misdiagnosed with polycystic ovary syndrome (PCOS). Subsequent work-up for primary infertility revealed idiopathic hypogonadotropic hypogonadism (IHH). She progressively developed insulin-dependent diabetes mellitus and life-threatening sudden heart failure, necessitating an urgent cardiac transplant. Genetic evaluation confirmed JH (type 2A), identifying a homozygous likely pathogenic variant in the hemojuvelin (*HJV*) gene c.1006G > T (p.Gly336Ter), reported in only 4 cases within the Indian population. This case underscores a critical clinical lesson: the simple failure to perform a routine, inexpensive ferritin test can allow JH to slip until it causes fatal multi-organ damage. We conclude that any young patient presenting with type D PCOS (non-hyperandrogenic), IHH, young-onset heart failure, or atypical diabetes must be screened for iron overload even in the absence of classical symptoms to prevent irreversible consequences. Early genetic testing should be considered.

## Introduction

Hereditary hemochromatosis (HH) is an autosomal recessive disorder characterized by the pathological accumulation of iron within vital organs, which eventually leads to progressive multi-organ failure [1]. Pathogenic variants in a number of genes regulating the hepcidin–ferroportin axis underlie this disease. The most common form of HH is caused by a pathogenic variant in the *HFE* gene (type 1 HH), which is a milder form and rarely causes cardiac or endocrine failure. Three rare non-HFE-related forms have been identified: type 2 juvenile hemochromatosis (JH), due to a pathogenic variant in the hemojuvelin (*HJV*) gene (type 2A, ∼90% of JH cases) or hepcidin antimicrobial peptide (*HAMP*) gene (type 2B); type 3, from transferrin receptor-2 (*TFR2*) pathogenic variant; type 4, from ferroportin (*SLC40A1*) pathogenic variant with autosomal dominant inheritance; and type 5, from the H-Ferritin (*FTH1*) pathogenic variant. JH is a rarer and more severe variant of HH that typically presents between 10 and 30 years of age, causing early onset of endocrine failure (hypogonadotropic hypogonadism and diabetes mellitus), cardiomyopathy, and cirrhosis [2].

## Case presentation

A 26-year-old female presented with primary infertility after 2 years of cohabitation. On evaluation, she was diagnosed with idiopathic hypogonadotropic hypogonadism (IHH). There was no history suggestive of other pituitary hormone deficiencies, visual field defects, or anosmia.

Family history was unremarkable. Further questioning revealed that she attained menarche at 13 years of age and had regular menstrual cycles until age 18, when she developed oligomenorrhea. There was no history of weight gain, hirsutism, acne, galactorrhea, proximal muscle weakness, easy fatigability, or cold intolerance. Based on her history of oligomenorrhea, ultrasound findings, and a withdrawal bleed after progesterone, she was initially diagnosed with polycystic ovary syndrome (PCOS), a common etiology. She did not seek further medical consultation thereafter, as she had no other associated symptoms except for irregular cycles, for which she took combined oral contraceptives (COCs) intermittently.

For infertility, she was started on gonadotropin therapy, and intrauterine insemination was performed. She achieved a successful twin delivery, and the postnatal period was uneventful. Six months postpartum, she consulted a physician for excessive fatigue, poor weight gain, and low mood, which significantly affected her quality of life. Following investigations, these symptoms were attributed to postpartum depression and nutritional deficiencies such as anemia, vitamin D deficiency; she was subsequently started on antidepressants, supplements, and COCs. She noted improvement in her mood, fatigability, and weight, leading her to continue her medications with the exception of the COCs. Two years later, while being evaluated for dry eyes, she was diagnosed with antibody-negative insulin-dependent diabetes mellitus. Within 5 months, she presented with sudden-onset orthopnea. An echocardiogram revealed dilated cardiomyopathy (DCM) with severely reduced ejection fraction of 18% to 20%. There was no prior history of chest pain, palpitations, paroxysmal nocturnal dyspnea, or pedal edema. Due to rapid deterioration of her cardiac function, she underwent an urgent cardiac transplant.

## Diagnostic assessment

The initial physical and clinical examination of the patient was within normal limits. A hormonal evaluation during the infertility work-up revealed a biochemical profile consistent with hypogonadotropic hypogonadism, specifically showing a serum luteinizing hormone (LH) of 0.9 mIU/mL (reference range, 0.50-16.90 mIU/mL), follicle-stimulating hormone (FSH) of 2.7 mIU/mL (reference range, 1.50-9.10 mIU/mL), and low estradiol of 11 pg/mL (SI: 40.38 pmol/L) (reference range, 31-90 pg/mL [SI: 114-330 pmol/L]). Other pituitary axes appeared normal; serum prolactin, cortisol, free thyroxine (FT4), thyroid-stimulating hormone (TSH), insulin-like growth factor (IGF-1), and plasma adrenocorticotropic hormone (ACTH) levels, all were within normal limits (Table 1).

**Table 1:** Laboratory endocrine evaluation at baseline

Laboratory test	International system (and conventional) units	Normal range
LH	0.9 IU/L (0.9 mIU/mL)	0.50-16.90 IU/L (0.50-16.90 mIU/mL)
FSH	2.7 IU/L (2.7 mIU/mL)	1.50-9.10 IU/L (1.50-9.10 mIU/mL)
Estradiol	40.38 pmol/L (11 pg/mL)	114-330 pmol/L (31-90 pg/mL)
Prolactin	7.9 µg/L (7.9 ng/mL)	2.10-17.70 µg/L (2.10-17.70 ng/mL)
FT4	10.3 pmol/L (0.8 ng/dL)	7.46-21.2 pmol/L (0.58-1.64 ng/dL)
TSH	1.29 mIU/L (1.29 µIU/mL)	0.55-4.78 mIU/L (0.5-4.78 µIU/mL)
Cortisol	496 nmol/L (18 µg/dL)	118.7-618.2 nmol/L (4.30-22.40 µg/dL)
ACTH	5.94 pmol/L (27 pg/mL)	<10.12 pmol/L (<46 pg/mL)
IGF-1	23.8 nmol/L (182 ng/mL)	10.9-33.9 nmol/L (83.6-259 ng/mL)
HbA1c	40 mmol/mol (5.8%)	<39 mmol/mol (<5.7%)

Abbreviations: ACTH, adrenocorticotropin; FSH, follicle-stimulating hormone; FT4, free thyroxine; HbA1c, glycated hemoglobin; IGF-1, insulin-like growth factor 1; LH, luteinizing hormone; TSH, thyrotropin.

A gonadotropin-releasing hormone stimulation test was performed with triptorelin (0.1 mg subcutaneously) as per institutional protocol; peak LH was 4.4 mIU/mL and FSH was 5.1 mIU/mL at 3 hours, and estradiol was 27 pg/mL (SI: 99.1 pmol/L) at 24 hours. The response was nonstimulable, as peak LH was below the minimum cutoff of 5 mIU/mL. Pituitary magnetic resonance imaging (MRI) was normal. Hence, a diagnosis of IHH was made. Hemochromatosis was not considered as an etiology of IHH because of the absence of classical symptoms, lack of significant family history, and rarity of this condition, especially in the Indian population. Furthermore, as fertility treatment was the primary focus at that time, she was not evaluated further.

When investigated for dry eyes, the patient was found to have a random plasma glucose level of 400 mg/dL (SI: 22.2 mmol/L) (reference range, 140 mg/dL [SI: <7.8 mmol/L]) and a glycated hemoglobin (HbA1c) level of 10.8% (95 mmol/mol) (reference range, <5.7% [SI: <39 mmol/mol]) without ketoacidosis (Table 1). An autoantibody panel for type 1 diabetes mellitus was negative, with an undetectable C-peptide level. Differential diagnoses, including pancreatic diabetes, antidepressant-induced hyperglycemia, or a rare viral etiology, were considered, and a contrast-enhanced computed tomography of the abdomen was planned. However, within 5 months, she developed DCM and underwent an urgent transplant. This strengthened the clinical suspicion of either a viral-induced etiology or a missed atypical postpartum cardiomyopathy, with chronic stress potentially contributing to the development of her diabetes mellitus. Histopathological analysis of the explanted cardiac tissue was not pursued, as hemochromatosis had not yet been considered.

The diagnostic challenge in this case resurfaced 1 year following the cardiac transplant, when blood glucose levels failed to improve, and mild transaminitis persisted, along with significant fatigue and poor weight gain.

Whole-exome sequencing was performed to explore an underlying unified etiology, identifying a homozygous likely pathogenic variant, c.1006G > T (p.Gly336Ter), in the *HJV* gene (OMIM #602390; ClinVar: VCV000560162). This likely pathogenic variant, which has been observed only in the Indian population, confirmed a diagnosis of JH type 2A. Subsequent laboratory testing revealed a massively elevated serum ferritin level of 4296 ng/mL (SI: 9653.3 pmol/L) (reference range, 10.00-291.00 ng/mL [SI: 22.47-653.93 pmol/L]) (Table 2).

**Table 2:** Laboratory evaluation

Laboratory test	International system (and conventional) units	Normal range
Ferritin	9653.3 pmol/L (4296 ng/mL)	22.47-653.93 pmol/L (10.00-291.00 ng/mL)
Iron	48.15 µmol/L (269 µg/dL)	5.91-34.55 µmol/L (33-193 µg/dL)
Alanine aminotransferase	1.31 µkat/L (79 U/L)	0-0.58 µkat/L (<35 U/L)
Aspartate aminotransferase	0.99 µkat/L (60 U/L)	0-0.83 µkat/L (<50 U/L)
Gamma-glutamyl transferase	1.72 µkat/L (104 U/L)	0.12-0.84 µkat/L (7-50 U/L)
25(OH)D	51.7 nmol/L (20.7 ng/mL)	75-250 nmol/L (30-100 ng/mL)
Calcium	2.32 mmol/L (9.3 mg/dL)	2.20-2.55 mmol/L (8.8-10.2 mg/dL)
Phosphorus	1.49 mmol/L (4.6 mg/dL)	1.13-1.71 mmol/L (3.5-5.3 mg/dL)
Alkaline phosphatase	1.24 µkat/L (74 U/L)	0.50-2.00 µkat/L (30-120 U/L)
CTX	1103.6 pmol/L (1270 pg/mL)	130.4-551.8 pmol/L (150-635 pg/mL)

Abbreviations: CTX, C-terminal telopeptide of type I collagen; 25(OH)D, 25-hydroxyvitamin D.

Retrospective histopathological examination of the explanted cardiac tissue, performed following the genetic diagnosis, demonstrated extensive myocardial iron deposition and associated interstitial fibrosis, substantiating the extent of iron overload (Fig. 1). Advanced imaging via abdominal MRI provided further confirmation of parenchymal iron overload, showing diffuse hypointense signals on T1 and T2 weighted images in the liver and a diffuse T1 hypointense signal in the pancreas (Fig. 2).

**Figure 1:**
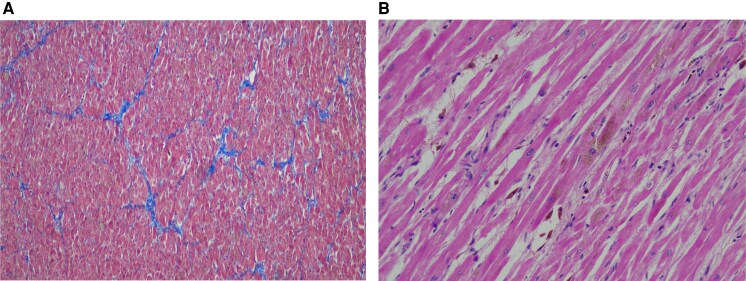
Histopathological examination of the explanted cardiac tissue in a patient with juvenile hemochromatosis shows (A) interstitial and replacement myocardial fibrosis on Masson trichrome stain (×10) and (B) coarse brown hemosiderin deposits within cardiomyocytes on hematoxylin and eosin stain (×20). These findings confirm iron overload–driven myocardial injury and chronic remodeling. Early serum ferritin screening here might have allowed timely chelation or phlebotomy and prevented progression to end-stage cardiomyopathy requiring transplantation.

**Figure 2:**
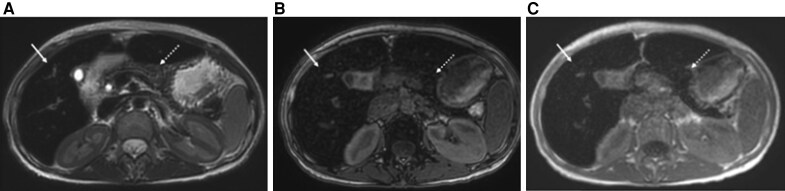
Abdominal MRI performed to assess parenchymal iron burden shows axial T2-weighted (A), out-of-phase T1 (B), and in-phase T1 (C) sequences. Diffusely decreased signal intensity is present in the liver (solid arrow) and pancreas (dashed arrow) across all sequences, with progressive signal loss on in-phase relative to out-of-phase T1, consistent with hepatic and pancreatic iron deposition. Splenic signal is preserved, a pattern characteristic of primary hemochromatosis. Qualitative MRI assessment of parenchymal iron burden is a noninvasive tool that can confirm multi-organ iron deposition before irreversible end-organ damage occurs. Its early use in young patients with unexplained hypogonadism or atypical diabetes could facilitate timely diagnosis and intervention.

## Treatment

Diabetes mellitus was managed with a basal-bolus insulin regimen. Following her cardiac transplant, she was started on tacrolimus (4.5 mg twice daily), prednisolone (5 mg daily), and mycophenolate (360 mg at 8.00 Am and 180 mg at 8.00 Pm). Due to highly elevated ferritin levels, iron chelation therapy was promptly initiated with oral deferasirox at a dose of 1500 mg once daily. Phlebotomy was deferred due to concurrent anemia.

After 1 year of therapy, the ferritin level decreased significantly (Table 3). For the management of hypogonadism, a low dose of estradiol valerate (1 mg) was started. To address osteoporosis, the patient was started on calcium (500 mg twice daily), cholecalciferol (60 000 IU once monthly), and annual intravenous injection zoledronate (5 mg).

**Table 3:** Trends of ferritin levels

Units	0 month	1st month	3rd month	5th month	7th month	8th month	11th month	15th month	18th month
SI (pmol/L)	9653.3	8002.2	6310.1	3489.8	3370.8	3060.7	3150.6	2042.7	1528.1
Conventional (ng/mL)	4296	3561	2808	1553	1500	1362	1402	909	680

## Outcome and follow-up

Two years post-cardiac transplant, the patient's cardiac status remains normal with an ejection fraction of 60%. Iron chelation therapy, initiated 1 year previously, is being gradually titrated based on ferritin levels; trends are summarized in Table 3. One year after transplant, she noted hyperpigmentation and desquamation of the skin. Blood glucose levels remain well-controlled on a basal-bolus insulin regimen, and liver function tests are being closely monitored because of mild transaminitis.

Regarding bone health, laboratory evaluation revealed a serum 25-hydroxyvitamin D [25(OH)D] level of 20.7 ng/mL (SI: 51.7nmol/L) (reference range, 30-100 ng/mL [SI: 75-250 nmol/L]). The serum marker of bone resorption, C-terminal telopeptide of type I collagen (CTX), was elevated at 1270 pg/mL (SI: 1103 pmol/L) (reference range, 150-635 pg/mL [SI: 130.4-551.8 pmol/L]) (Table 2). Bone density evaluation using dual-energy x-ray absorptiometry (DXA: GE Lunar) revealed a *Z*-score of −3.8 and a *T*-score of −4.5 at the lumbar spine (Table 4). Over the last 6 months, she has reported significant arthralgia and neuropathic symptoms (numbness and pain) in both the upper and lower limbs.

**Table 4:** DXA report (GE lunar)

Site	BMD (g/cm^2^)	*T*-score	*Z*-score
L1-L4 (lumbar spine)	0.635	−4.5	−3.8
Left total hip	0.508	−4.0	−3.3
Left neck of femur	0.588	−3.2	−2.6
Distal 1/3 radius	0.621	−2.9	−2.9

Abbreviations: BMD, bone mineral density; DXA, dual-energy x-ray absorptiometry.

## Discussion

JH is a rare form of iron overload that often presents with “clinical perplexity” due to its rarity and frequent lack of classical features or family history. Because of early onset and rapid iron overload associated with this condition, it results in complications such as hypogonadotropic hypogonadism, cardiomyopathy, and diabetes, all of which increase mortality risk [2, 3].

Iron deposition in the pituitary gonadotrophs is the primary cause of hypogonadism, which is the most common early manifestation of JH. It affects 67% to 96% of patients and often presents as amenorrhea or oligomenorrhea in females and decreased facial hair or reduced libido in males [4]. In contrast, in the more common type 1 HH, hypogonadism is uncommon; its presence usually indicates advanced disease [5]. Early diagnosis and treatment of iron overload, including phlebotomy, may restore gonadotropin function, underscoring the importance of timely recognition and intervention [6].

Diabetes is another common endocrinopathy in JH, with a prevalence of 13% to 23% of affected individuals [7]. Impaired glucose tolerance is even more common, reported in up to 58% of individuals with type 2 JH, compared with 27% and 9.1% in types 1 and 3, respectively [8]. In JH, addressing iron overload may improve glucose tolerance, especially if intervention occurs before significant organ damage or complications develop [9].

Our patient also developed DCM, a life-threatening complication frequently seen in JH. Excessive iron deposition induces oxidative stress and cardiomyocyte injury, reducing cardiac function and significantly increasing mortality risk [10]. Early recognition of cardiac involvement is essential, as timely iron chelation or phlebotomy may slow disease progression and help preserve cardiac function; in some cases, cardiac transplantations could even be prevented.

On follow-up, severe osteoporosis was detected, for which she was treated with zoledronic acid. The possible mechanisms of osteoporosis seen in JH are thought to be related to hypogonadism and direct iron toxicity to the bones [11], both of which were operative in our patient.

Of note, JH is very rare in Asian populations. In India, only 6 cases have been identified to date, of which 4 had a particular *HJV* gene pathogenic variant (p.Gly336Ter). This finding was mirrored in our patient, suggesting ethnicity may play a role [12, 13].

This case further highlights that hemochromatosis should not be dismissed on the basis of ethnicity or atypical presentation. Currently, routine screening for JH in the general population is not recommended. However, for patients presenting with unexplained symptoms such as secondary amenorrhea, heart failure, atypical diabetes, or fatigue, evaluation for iron overload should be considered. Classical symptoms such as skin hyperpigmentation and arthralgia may be absent early in the disease or are less common in *non-HFE* hemochromatosis. Therefore, maintaining a high index of suspicion is crucial. Initial tests, such as serum ferritin and transferrin saturation, are cost-effective and should be performed, followed by confirmatory genetic testing if results are abnormal.

As the management was done at different centers, each presentation, including oligomenorrhea, IHH, atypical diabetes, and ultimately fatal cardiomyopathy, was considered in isolation. As a result, the unifying diagnosis could only be established after an irreversible endpoint had been reached.

## Learning points

- All patients diagnosed with IHH should undergo routine serum ferritin testing to rule out iron overload.
- High index of clinical suspicion is vital in atypical presentations of hemochromatosis, even when classical clinical features are absent.
- Early diagnosis is crucial, as timely intervention can prevent irreversible organ damage.
- In young patients presenting with pancreatic diabetes, a hemochromatosis workup should be considered as part of the diagnostic evaluation.
- Genetic testing should be pursued when clinical features are incongruent with more common diseases.

## Data Availability

Original data generated and analyzed during this study are included in this published article.
